# Personalised prediction of maintenance dialysis initiation in patients with chronic kidney disease stages 3–5: a multicentre study using the machine learning approach

**DOI:** 10.1136/bmjhci-2023-100893

**Published:** 2024-04-27

**Authors:** Anh Trung Hoang, Phung-Anh Nguyen, Thanh Phuc Phan, Gia Tuyen Do, Huu Dung Nguyen, I-Jen Chiu, Chu-Lin Chou, Yu-Chen Ko, Tzu-Hao Chang, Chih-Wei Huang, Usman Iqbal, Yung-Ho Hsu, Mai-Szu Wu, Chia-Te Liao

**Affiliations:** 1 Nephro-Urology and Dialysis Center, Bach Mai Hospital, Hanoi, Vietnam; 2 Clinical Data Center, Office of Data Science, Taipei Medical University, Taipei, Taiwan; 3 Clinical Big Data Research Center, Taipei Medical University Hospital, Taipei, Taiwan; 4 Research Center of Health Care Industry Data Science, College of Management, Taipei Medical University, Taipei, Taiwan; 5 International PhD program of Biotech and Healthcare Management,College of Management, Taipei Medical University, Taipei, Taiwan; 6 University Medical Center, Ho Chi Minh City, Vietnam; 7 Department of Internal Medicine, Hanoi Medical University, Hanoi, Vietnam; 8 Division of Nephrology, Department of Internal Medicine, Shuang Ho Hospital, Taipei Medical University, New Taipei City, Taiwan; 9 Division of Nephrology, Department of Internal Medicine, School of Medicine, College of Medicine, Taipei Medical University, Taipei, Taiwan; 10 TMU-Research Center of Urology and Kidney (TMU-RCUK), Taipei Medical University, Taipei, Taiwan; 11 Division of Nephrology, Department of Internal Medicine, Hsin Kuo Min Hospital, Taipei Medical University, Taoyuan City, Taiwan; 12 Division of Nephrology, Department of Medicine, Tri-Service General Hospital, National Defense Medical Center, Taipei, Taiwan; 13 Division of Cardiovascular Surgery, Department of Surgery, Shuang Ho Hospital, Taipei Medical University, New Taipei City, Taiwan; 14 Graduate Institute of Biomedical Informatics, College of Medical Science and Technology, Taipei Medical University, Taipei, Taiwan; 15 International Center for Health Information Technology, College of Medical Science and Technology, Taipei Medical University, Taipei, Taiwan; 16 School of Population Health, Faculty of Medicine and Health, University of New South Wales (UNSW), Sydney, New South Wales, Australia; 17 Global Health & Health Security Department, College of Public Health, Taipei Medical University, Taipei, Taiwan

**Keywords:** Machine Learning, Primary Health Care, Preventive Medicine, Patient Outcome Assessment, Electronic Data Processing

## Abstract

**Background:**

Optimal timing for initiating maintenance dialysis in patients with chronic kidney disease (CKD) stages 3–5 is challenging. This study aimed to develop and validate a machine learning (ML) model for early personalised prediction of maintenance dialysis initiation within 1-year and 3-year timeframes among patients with CKD stages 3–5.

**Methods:**

Retrospective electronic health record data from the Taipei Medical University clinical research database were used. Newly diagnosed patients with CKD stages 3–5 between 2008 and 2017 were identified. The observation period spanned from the diagnosis of CKD stages 3–5 until the maintenance dialysis initiation or a maximum follow-up of 3 years. Predictive models were developed using patient demographics, comorbidities, laboratory data and medications. The dataset was divided into training and testing sets to ensure robust model performance. Model evaluation metrics, including area under the curve (AUC), sensitivity, specificity, positive predictive value, negative predictive value and F1 score, were employed.

**Results:**

A total of 6123 and 5279 patients were included for 1 year and 3 years of the model development. The artificial neural network demonstrated better performance in predicting maintenance dialysis initiation within 1 year and 3 years, with AUC values of 0.96 and 0.92, respectively. Important features such as baseline estimated glomerular filtration rate and albuminuria significantly contributed to the predictive model.

**Conclusion:**

This study demonstrates the efficacy of an ML approach in developing a highly predictive model for estimating the timing of maintenance dialysis initiation in patients with CKD stages 3–5. These findings have important implications for personalised treatment strategies, enabling improved clinical decision-making and potentially enhancing patient outcomes.

**WHAT IS ALREADY KNOWN ON THIS TOPIC**

- Early prediction of dialysis initiation in patients with chronic kidney disease (CKD) is invaluable for tailoring personalised treatment plans. Despite several prediction models that have been developed, they almost lack sufficient accuracy and fail to include several crucial factors related to CKD progression.

**WHAT THIS STUDY ADDS**

- Developing a machine learning predictive model that incorporates comprehensive clinical data and standardises the selection of the index date may lead to even more accurate predictions. So far, this study has involved the largest patient enrolment and the broadest range of clinical parameters.

**HOW THIS STUDY MIGHT AFFECT RESEARCH, PRACTICE OR POLICY**

- These findings enable highly accurate estimation of the timing for initiating maintenance dialysis in patients with CKD stages 3–5. As a result, they have significant implications for personalised treatment strategies, facilitating improved clinical decision-making and potentially enhancing patient outcomes.

## Introduction

Chronic kidney disease (CKD) and end-stage renal disease (ESRD) are significant global health problems that burden healthcare systems worldwide. In Taiwan, the overall prevalence of CKD was 8.2%, and the incidence of treated ESRD was 529 per million population, according to the US Renal Data System annual report published in 2021.^1^ International guidelines recommend referring patients with CKD to nephrology for pre-ESRD care upon reaching an advanced stage to improve the quality of care and reduce costs.^2 3^ One critical component of pre-ESRD care is counselling patients on choosing kidney replacement therapy (KRT) following shared decision-making. It may involve preparing vascular access for haemodialysis (HD) at least 6 months before HD initiation, placing a peritoneal dialysis (PD) catheter at least 2 weeks before PD initiation, or identifying suitable donors for pre-emptive kidney transplantation before dialysis is required to replace failing kidney function. Therefore, personalising the timing of referral for maintenance dialysis initiation is essential for each patient. However, early personalised estimation of the optimal timing for maintenance dialysis initiation presents a significant challenge for patients with CKD. This decision relies not only on the glomerular filtration rate (GFR) level but also on symptoms of uraemia syndrome and the ability to manage complications such as electrolyte imbalance, acid–base disturbances and fluid overload through medical treatment.^3–5^

Numerous traditional and artificial intelligence (AI) models have been developed and evaluated to estimate the duration until the initiation of KRT among patients with CKD.^6–10^ Among these models, machine learning (ML) approaches that use complex computer algorithms are effective in identifying the most critical factors and developing predictive models with superior performance.^11 12^ However, existing models rely primarily on laboratory data and comorbidities to inform their analyses and predictions, neglecting important indicators of CKD progression such as an annual decline in GFR, proteinuria and medication use. Furthermore, the timing (index date) chosen for these models is heterogeneous due to the diverse stages of CKD represented in the patient cohorts, resulting in relatively low predictive power. Therefore, developing an accurate and reliable predictive model that incorporates comprehensive clinical data and standardises index date selection is crucial to improving personalised decision-making for patients with CKD.

In this study, we aim to develop and validate an ML model for early personalised prediction of maintenance dialysis initiation within 1-year and 3-year timeframes among patients with CKD stages 3–5 using a multicentre longitudinal cohort.

## Materials and methods

### Study data source

We conducted a retrospective analysis of the Taipei Medical University (TMU) clinical research database (TMUCRD), which comprises comprehensive medical claims data for patients across three affiliated hospitals: TMU Hospital (TMUH), Wan Fang Hospital (WFH) and Shuang Ho Hospital (SHH). The TMUCRD encompasses structured and unstructured data for over 4 million patients, spanning the period from 1998 to 2021. All data were fully anonymised prior to analysis, with patients’ identity codes and medical facility information scrambled to ensure patient privacy. This study was authorised by the joint institutional review board (IRB) committee of TMU (IRB#: N202105032).

### Study population

**Figure 1:**
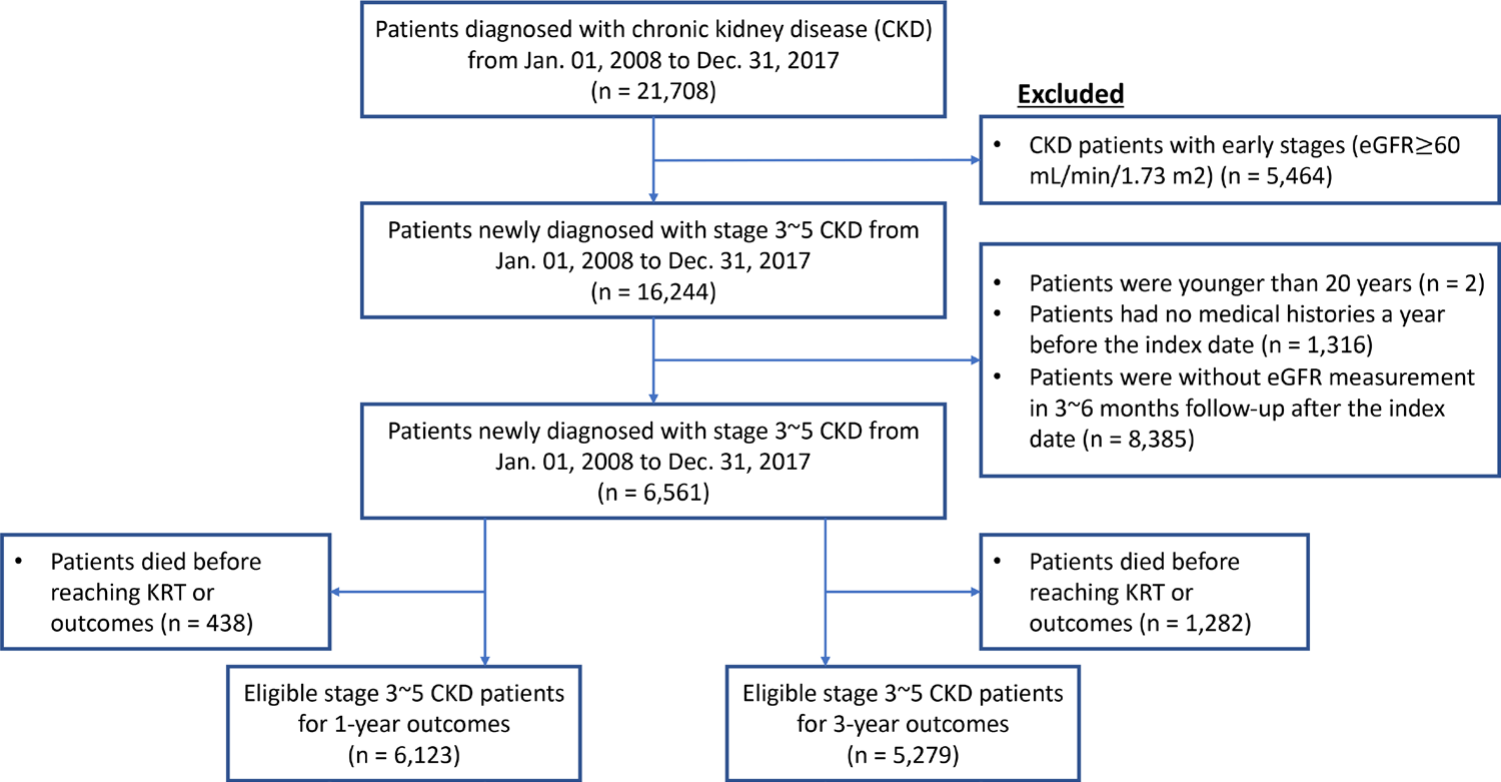
Enrollment process of the first study. The index date is the point of time at which patients with CKD were first diagnosed with stages 3–5. CKD, chronic kidney disease; eGFR, estimated glomerular filtration rate; KRT, kidney replacement therapy.

We identified patients diagnosed with CKD between 1 January 2008 to 31 December 2017, based on the International Classification of Disease, ninth revision (ICD-9) code 585 and 10th revision (ICD-10) code N18. First-time patients diagnosed with CKD stages 3–5 and who had not undergone KRT, defined as the index date, were included in the study. We confirmed the diagnosis based on the G-stages, in which the estimated glomerular filtration rate (eGFR) was calculated using the CKD-EPI creatinine equation.^13^ To define correctly the stage of CKD, only patients who had been diagnosed with CKD prior to the index dates and had their eGFRs observed within 3–6 months after the index date were included. Patients who were younger than 20 years at the time of diagnosis of CKD stages 3–5 or underwent pre-emptive kidney transplantation were excluded from the study (figure 1).

### Observation period

Patients were followed up from the time of stages 3–5 CKD diagnosis (ie, the index date), and their data were censored at the start of maintenance dialysis, loss to follow-up, termination of insurance or the end of the study period (ie, 3 years from the index date). Additionally, patients who died before initiating KRT during the follow-up were excluded from the study.

### Outcome measurement

The primary outcome of this study was the initiation of maintenance dialysis among patients with CKD stages 3–5. The initial dialysis point was defined as the first day of maintenance dialysis treatment (eg, the first day of long-term HD, the day of catheter insertion for PD or the first day of PD) based on the procedure-related codes under Taiwan’s National Health Insurance (online supplemental table S1). The patients who underwent maintenance dialysis were defined as ‘receiving dialysis’ and others as ‘living without dialysis’.

10.1136/bmjhci-2023-100893.supp1 Supplementary data

### Variables and data processing

Patient demographics, comorbidities, medications and laboratory data were collected from the database within 1 year before the diagnosis date. The major comorbidities were identified using diagnostic codes (ICD-9 and ICD-10) from outpatient and inpatient databases. The analysis included all diseases listed in the Charlson Comorbidity Index (CCI),^14^ along with additional conditions such as essential hypertension, glomerular diseases, lipid metabolism disorders and septicaemia. These diseases were considered confirmed if at least one outpatient or inpatient visit was documented within 1 year prior to the diagnosis date.

The TMUCRD contains comprehensive information on prescribed medications from three affiliated hospitals. Patients’ medication prescription claims were tracked using the Anatomical Therapeutic Chemical codes for 1 year preceding the diagnosis date of CKD stages 3–5 (online supplemental table S2).

We also retrieved some routine blood tests from laboratory datasets, including haemoglobin (Hgb), white blood cells (WBC), neutrophils, platelets (PLT), blood urea nitrogen (BUN), creatinine (CREA), cholesterol (CHOL), triglyceride (TG), albumin, calcium, phosphorus, sodium and potassium. The average value of each blood test was calculated based on the results collected within 1 year before the diagnosis date. Blood tests that had over 50% missing values were excluded from the analysis. To manage the missing continuous features, we used the Multiple Imputation by Chained Equations method to fill in these gaps in the data.^15^ The eGFR was measured at the diagnosis date (the baseline eGFR) and compared with the previous eGFR, from which the decline of eGFR was calculated using the formula:

The decline of eGFR = (Previous eGFR–Baseline eGFR)/the day interval

In addition, we also collected urine tests and classified albuminuria based on albuminuria categories according to the Kidney Disease Improving Global Outcomes (KDIGO) classification^13^ (online supplemental table S3). Patients with missing values on albuminuria were defined as the group ‘unknown’.

### Modelling

The classification models were used to predict the initial dialysis, including logistic regression, linear discriminant analysis, gradient boosting machine (GBM), light GBM, AdaBoost, random forest (RF), extreme gradient boosting machine (Xgboost) and artificial neural networks (ANN). A detailed description of different models and their parameters is shown in online supplemental appendix S1.

### Model training and testing

To ensure robust model development and account for sample selection bias, we divided the dataset into two parts: the training set and the test set. The training set consisted of patient data from two hospitals, TMUH and WFH, and was used for model development. To evaluate the performance of different ML models and estimate generalisation errors, we applied the stratified fivefold cross-validation method within the training set. This involved dividing the patients into five groups while ensuring that each group represented a proportional distribution of patient characteristics. Each group was then used as the internal validation set for one of the five replications. On the other hand, the test set comprised patient data obtained from SHH and served as an independent dataset for external model validation.

### Statistical analysis and evaluation of model performance

Continuous variables were provided as the mean±SD, and categorical variables were provided as absolute (n) and relative (%) frequency; it is described in table 1.

**Table 1:** Demographic characteristics of patients with CKD stages 3–5

	1-year prediction model*		3-year prediction model*	
	Training (n=4496)	Testing (n=1627)	Training (n=3879)	Testing (n=1400)
Age, years, N (%)				
Age <65	1235 (27.5)	478 (29.4)	1185 (30.5)	451 (32.2)
Age ≥65	3261 (72.5)	1149 (70.6)	2694 (69.5)	949 (67.8)
Mean (SD)	72.1 (13.4)	71.3 (13.0)	70.8 (13.3)	70.1 (12.9)
Median (IQR)	74(63 - 82)	73(62 - 81)	73(62 - 81)	71(61 - 80)
Gender, female, N (%)	1872 (41.6)	656 (40.3)	1610 (41.5)	564 (40.3)
Baseline G-stages, N (%)				
G3a	1196 (26.6)	314 (19.3)	1067 (27.5)	270 (19.3)
G3b	1328 (29.5)	390 (24.0)	1142 (29.4)	319 (22.8)
G4	1219 (27.1)	462 (28.4)	995 (25.7)	387 (27.6)
G5	753 (16.7)	461 (28.3)	675 (17.4)	424 (30.3)
Baseline eGFR, mL/min/1.73 m^2^, mean (SD)	32.2 (15.5)	27.2 (15.8)	32.4 (15.7)	26.7 (16.0)
Decline of eGFR, mL/min/1.73 m^2^, mean (SD)	−0.173 (1.43)	−0.127 (0.75)	−0.166 (1.42)	−0.104 (0.75)
Patients with maintenance dialysis, N (%)††	341 (7.6)	216 (13.3)	752 (19.4)	403 (28.8)
Comorbidities, N (%)				
Diabetes mellitus	2308 (51.3)	850 (52.2)	1994 (51.4)	733 (52.4)
Essential hypertension	2747 (61.1)	782 (48.1)	2385 (61.5)	657 (46.9)
Glomerular diseases	1055 (23.5)	395 (24.3)	951 (24.5)	345 (24.6)
Septicaemia	203 (4.5)	143 (8.8)	157 (4.0)	101 (7.2)
Malignant neoplasm	460 (10.2)	102 (6.3)	356 (9.2)	75 (5.4)
Disorders of lipoid metabolism	2124 (47.2)	554 (34.1)	1897 (48.9)	488 (34.9)
Ischaemic heart disease	1541 (34.3)	534 (32.8)	1302 (33.6)	440 (31.4)
Cardiac dysrhythmias	739 (16.4)	187 (11.5)	591 (15.2)	145 (10.4)
Congestive heart failure	918 (20.4)	446 (27.4)	728 (18.8)	366 (26.1)
Cerebrovascular disease	912 (20.3)	326 (20.0)	717 (18.5)	258 (18.4)
Peripheral vascular disease	202 (4.5)	52 (3.2)	165 (4.3)	41 (2.9)
Chronic pulmonary disease	625 (13.9)	218 (13.4)	496 (12.8)	162 (11.6)
Chronic liver disease	391 (8.7)	111 (6.8)	333 (8.6)	91 (6.5)
CCI, N (%)				
CCI <3	1046 (23.3)	367 (22.6)	975 (25.1)	350 (25.0)
CCI ≥3	3450 (76.7)	1260 (77.4)	2904 (74.9)	1050 (75.0)
Mean (SD)	3.84 (1.75)	3.80 (1.57)	3.72 (1.65)	3.67 (1.50)
Median (IQR)	4 (3–5)	4 (3–5)	3 (2–5)	3 (2.25–5)
Medications, N (%)				
Antacids	1256 (27.9)	481 (29.6)	1055 (27.2)	407 (29.1)
H2-receptor antagonists	745 (16.6)	261 (16.0)	622 (16.0)	212 (15.1)
Proton pump inhibitors	569 (12.7)	335 (20.6)	451 (11.6)	273 (19.5)
Laxatives	1484 (33.0)	552 (33.9)	1177 (30.3)	428 (30.6)
Insulins and analogues	906 (20.2)	364 (22.4)	763 (19.7)	314 (22.4)
Sulfonylureas	838 (18.6)	285 (17.5)	754 (19.4)	254 (18.1)
Dipeptidyl peptidase-4 inhibitors	1028 (22.9)	349 (21.5)	904 (23.3)	300 (21.4)
Antiplatelets	2264 (50.4)	1024 (62.9)	1947 (50.2)	872 (62.3)
Vitamin B12 and folic acid	1370 (30.5)	181 (11.1)	1195 (30.8)	165 (11.8)
Organic nitrates	995 (22.1)	470 (28.9)	831 (21.4)	387 (27.6)
Diuretics	2031 (45.2)	789 (48.5)	1682 (43.4)	646 (46.1)
Purine derivatives	1567 (34.9)	629 (38.7)	1386 (35.7)	558 (39.9)
Beta-blocking agents	1894 (42.1)	739 (45.4)	1652 (42.6)	642 (45.9)
Calcium channel blockers	2381 (53.0)	889 (54.6)	2059 (53.1)	767 (54.8)
Agents acting on the renin-angiotensin system	2250 (50.0)	864 (53.1)	1974 (50.9)	748 (53.4)
Statins	1738 (38.7)	576 (35.4)	1547 (39.9)	515 (36.8)
Corticosteroids	802 (17.8)	323 (19.9)	665 (17.1)	250 (17.9)
Beta-lactam antibacterial	1535 (34.1)	657 (40.4)	1243 (32.0)	524 (37.4)
Non-steroids	1287 (28.6)	516 (31.7)	1113 (28.7)	431 (30.8)
Antigout preparations	1736 (38.6)	490 (30.1)	1519 (39.2)	424 (30.3)
Cough and cold preparations	1335 (29.7)	510 (31.3)	1074 (27.7)	410 (29.3)
Antihistamines	718 (16.0)	329 (20.2)	592 (15.3)	261 (18.6)
Laboratory tests, mean (SD)				
Haemoglobin, g/dL	113 (19.3)	110 (21.0)	114 (19.2)	110 (21.2)
White blood cells k/uL	7.55 (2.61)	7.94 (3.32)	7.52 (2.56)	7.90 (3.22)
Neutrophils, %	70.3 (8.74)	71.7 (9.55)	70.3 (8.50)	71.7 (9.22)
Platelets, mL	206 (65.4)	215 (75.4)	208 (64.3)	214 (71.4)
Blood urea nitrogen, mg/dL	37.3 (21.1)	42.2 (24.9)	37.2 (21.1)	42.9 (25.4)
Creatinine, mg/dL	2.45 (1.73)	3.06 (2.17)	2.49 (1.80)	3.18 (2.25)
Aspartate transferase, U/L	18.7 (17.9)	26.9 (24.7)	18.3 (17.2)	25.9 (20.4)
Cholesterol, mg/dL	179 (39.3)	184 (42.8)	180 (39.7)	185 (43.7)
Triglycerides, mg/dL	147 (103)	157 (273)	148 (106)	160 (289)
Albumin, g/dL	3.96 (0.43)	3.98 (0.20)	3.98 (0.42)	3.98 (0.20)
Calcium, mg/dL	8.98 (0.52)	8.96 (0.53)	8.99 (0.52)	8.96 (0.53)
Phosphorous, mg/dL	3.83 (0.62)	4.04 (0.75)	3.84 (0.64)	4.08 (0.77)
Sodium, mmol/L	139 (3.72)	138 (3.8)	139 (3.56)	138 (3.68)
Potassium, mmol/L	4.40 (0.60)	4.41 (0.68)	4.41 (0.59)	4.43 (0.68)
Albuminuria, N (%)				
A1	845 (18.8)	262 (16.1)	751 (19.4)	225 (16.1)
A2	651 (14.5)	183 (11.2)	565 (14.6)	158 (11.3)
A3	1494 (33.2)	679 (41.7)	1320 (34.0)	609 (43.5)
Unknown	1506 (33.5)	503 (30.9)	1243 (32.0)	408 (29.1)

*Study aimed to develop two prediction models that observed patients for 1 year and 3 years.

†Patients with KRT or maintenance dialysis is the outcome of the study.

CCI, Charlson Comorbidity Index; eGFR, estimated Glomerular filtration rate; G-stages, glomerular filtration rate stages; KRT, kidney replacement therapy.;

The area under the receiver operating characteristic (ROC) curve (AUC), accuracy, sensitivity (recall), specificity, positive predictive value (precision), negative predictive value and F1 score were computed to evaluate and compare the performance of all prediction models. The model with the highest AUC was selected as the best model through comparison using the external testing set. Furthermore, the impact of features in the best model was analysed using Shapley Additive exPlanations (SHAP) values.^16^

Data processing was conducted using SQL Server Management Studio V.18.6 (Redmond, Washington, USA), while model training and testing were performed using Python V.3.8.8 software (Wilmington, Detroit, USA) with scikit-learn V.1.1 (Paris, France).

## Results

### Data extraction

We identified 16 244 eligible patients with CKD stages 3–5 who were diagnosed for the first time in three TMU-affiliated hospitals from 1 January 2008 to 31 December 2017. Among those, we excluded 9703 patients due to being younger than 20, having no medical histories a year before the index date or lacking evidence for the diagnosis of CKD. Furthermore, we excluded 438 and 1282 patients for the 1-year and 3-year prediction models, respectively, who had died prior to undergoing maintenance dialysis. Finally, 6123 and 5279 patients who met all inclusion criteria were included in model development for 1-year and 3-year prediction performances, respectively (figure 1).

### Study population characteristics

The patients’ demographics, comorbidities, medications and laboratory data were summarised in table 1. The study population was over 65 years, with a mean (SD) ages of 71.7 (13.2) and 70.5 (13.1) years for the 1-year and 3-year prediction models, respectively. Most patients were male (59.1%) and had baseline G-stages of G3 (eg, 56% for the training set and 43% for the testing set). Patients had a high prevalence of comorbidities such as diabetes (51%), hypertension (61%), disorders of lipid metabolism (47%) and glomerular disease (24%). The outcome of initiation of maintenance dialysis was observed at 10.5% and 24.1% for the 1-year and 3-year prediction models, respectively.

### The performances of different prediction models

**Figure 2:**
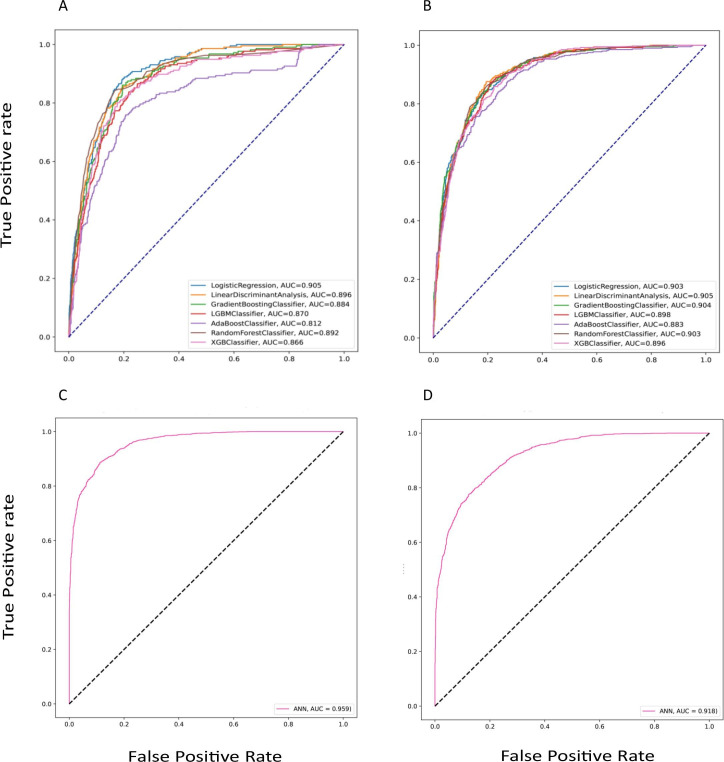
The performance of the prediction models in the testing dataset. (A and C) 1-year prediction with machine learning and ANN models; (B and C) 3-year prediction with machine learning and ANN models. ANN, artificial neural network; AUC, area under the receiver operating characteristic curve; LGBM, light gradient boosting machine; XGB, extreme gradient boosting.

The performances of different prediction models are shown in table 2. For a 1-year prediction of successful dialysis treatment, the highest AUC value of 0.96 was observed for the ANN model (ie, sensitivity, 0.88; specificity, 0.75; precision, 0.39 and F1 score, 0.6), followed by the GBM and RF models with an AUC value of 0.89. Likewise, the ANN model was performed with a better AUC value of 0.92 (ie, sensitivity, 0.87; specificity, 0.79; precision, 0.63 and F1 score, 0.73) than other ML models in the 3-year prediction of receiving maintenance dialysis. The ROC curves of varying prediction models for 1-year and 3-year successful dialysis treatment are shown in figure 2.

**Table 2:** Summary of different classification models

Classifiers	Training AUC	Testing AUC	Accuracy	Sensitivity	Specificity	Precision	F1 score
1-year prediction model performances							
Logistic regression	0.92	0.90	0.80	0.89	0.79	0.39	0.71
Linear discriminant analysis	0.92	0.90	0.80	0.87	0.79	0.39	0.67
Gradient boosting classifier	0.98	0.89	0.81	0.83	0.81	0.40	0.63
LGBM classifier	1.00	0.86	0.76	0.83	0.75	0.34	0.60
Ada Boost classifier	0.97	0.81	0.83	0.69	0.85	0.41	0.51
Random forest classifier	1.00	0.89	0.82	0.85	0.82	0.42	0.65
XGB classifier	1.00	0.87	0.82	0.80	0.82	0.41	0.63
ANN*	**0.99**	**0.96**	**0.89**	**0.88**	**0.75**	**0.39**	**0.60**
3-year prediction model performances							
Logistic regression	0.91	0.90	0.80	0.88	0.77	0.61	0.82
Linear discriminant analysis	0.92	0.91	0.83	0.86	0.82	0.66	0.81
Gradient boosting classifier	0.96	0.91	0.82	0.84	0.81	0.65	0.81
LGBM classifier	1.00	0.90	0.80	0.89	0.76	0.60	0.82
Ada Boost classifier	0.95	0.89	0.79	0.84	0.78	0.60	0.80
Random forest Classifier	1.00	0.90	0.80	0.88	0.77	0.61	0.82
XGB classifier	1.00	0.90	0.82	0.83	0.82	0.65	0.80
ANN*	**0.95**	**0.92**	**0.82**	**0.87**	**0.79**	**0.63**	**0.73**

*Best model based on AUC values.

.ANN, artificial neural network; AUC, area under the receiver operating characteristic curve; LGBM, light gradient boosting machine; XGB, extreme gradient boosting.

### Features importance

**Figure 3:**
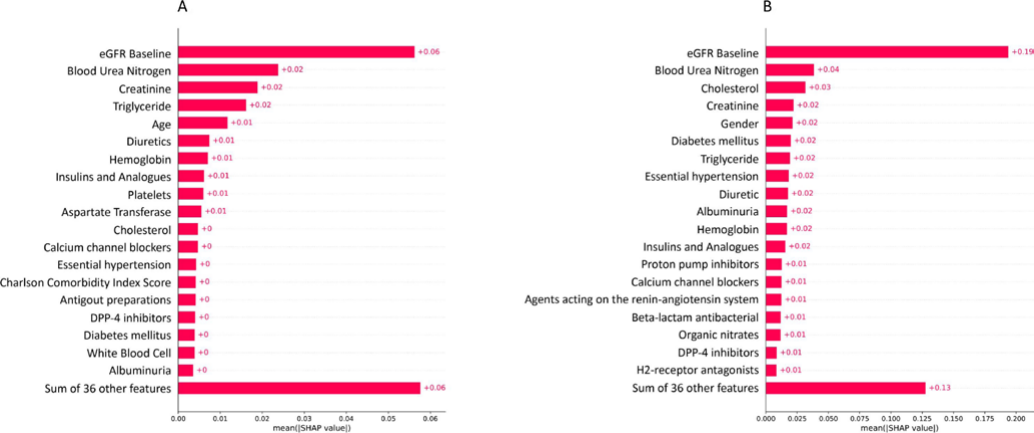
Feature importance of the ANN prediction model. (A) 1-year prediction model and (B) 3-year prediction model. ANN, artificial neural network; eGFR, estimated glomerular filtration rate; DPP-4, dipeptidyl peptidase

The lists of the top 20 important features that might impact the prediction model’s performance for 1-year and 3-year successful dialysis are shown in figure 3. The essential features of the 1-year and 3-year follow-up models were baseline eGFR, BUN, creatinine, triglyceride, age, gender, Hgb, CHOL, PLTs, albuminuria, diabetes disease, hypertension and related medications (eg, diuretics, insulin, dipeptidyl peptidase-4 and calcium channel blockers).

## Discussion

Our study findings demonstrated that ML classification models are well suited for a meaningful prediction of the initiation of maintenance dialysis in patients with CKD stages 3–5. The ANN method showed a better performance level for 1-year and 3-year prediction of dialysis commencement with a higher AUC (0.96 and 0.92), good sensitivity (0.88 and 0.87) and specificity (0.75 and 0.79).

In previous studies, AI models have been applied to predict CKD progression and start KRT. In 2015, Jamshid Norouzi *et al* used an adaptive neuro-fuzzy inference system to predict renal failure progression. Their model could accurately (>95%) predict the GFR for 6-month to 18-month intervals. However, only 465 patients with CKD were included in their study, and it was noted that proteinuria was not an important feature in their model.^8^ In 2019, Jing Xiao *et al* developed ML models to predict CKD progression. Their model used only the patient’s demographics and biochemical blood features, not features derived from a urinalysis. Besides, the predictive power of the model was not high (AUC: 0.873, sensitivity: 0.83 and specificity: 0.82).^17^ Another model was performed using only comorbidity data from 8492 patients to predict the onset of KRT, and their results were even lower (AUC, sensitivity and specificity were only 0.773, 0.623 and 0.781, respectively).^7^ Recently, Qiong Bai *et al* also conducted an ML model to predict the risk of ESRD. This model selected many important factors associated with the progression of CKD, including demographics, blood tests and comorbidities, but not proteinuria. However, the predictability has not improved compared with previous models.^18^ It could be explained by the fact that many patients in that study were in the early stages of CKD, resulting in a low percentage of those who progressed to ESRD when followed for a short period of time. The imbalance in the outcome can significantly affect the model’s predictive power.

In this study, we only focused on patients with CKD stages 3–5, and their risk of progression to ESRD is high. Hence, predicting the time of their dialysis commencement is very practical in our daily clinical care. Moreover, we carefully identified the model features associated with CKD progression and KRT based on the clinical setting and traditional logistic regression analysis. Forty-five significant prognostic factors were selected, including patient demographics, comorbidities, routine blood and urine tests and commonly used medications. Therefore, the predictive ability of our model has higher accuracy.

In further analysis of the ANN model, we also ranked all predictors according to their influence on the 1-year and 3-year models using SHAP values.^19^ Notably, several distinct features have been identified, respectively. For example, age, comorbidity (CCI score), PLT counts and WBC counts were important contributing factors in the 1-year prediction model, whereas gender and other medications such as proton pump inhibitors, beta-lactam antibacterial agents, organic nitrates and H2-receptor antagonists were relevant factors in the 3-year model. Common important factors identified in both models included eGFR at baseline, blood urea, serum creatinine and albuminuria (see figure 3). These are also key determinants for the risk classification of CKD according to the 2012 KDIGO guidelines.^13 20^ Other contributing factors in both models included serum Hgb level, TG or CHOL levels, hypertension, diabetes mellitus, diuretic use, antihypertensive agents and medications for controlling blood glucose levels (see figure 3). Anaemia typically develops during the course of CKD; a decrease in serum Hgb is significantly associated with the progression of CKD.^21 22^ Diabetic nephropathy is the leading cause of ESRD in adults.^23 24^ In patients with diabetic CKD, blood glucose levels are associated with poor outcomes such as serum creatinine doubling, ESRD and mortality, and intensive glycaemic control could reduce these risks.^25–29^ Additionally, several studies have demonstrated that certain levels of dyslipidaemia is independently associated with rapid renal progression, KRT, all-cause mortality and cardiovascular death in predialysis patients.^30–33^ Hypertension may occur early during the course of CKD and is related to a more rapid decline of kidney function, the development of cardiovascular disease and death in patients with CKD.^34 35^ Early intervention and tight control of blood pressure could lessen the risk of CVD and all-cause death in patients with and without CKD.^36 37^ Diuretics are an important part of guideline-directed medical therapy for patients with CKD with hypertension, oedema and hyperkalaemia.^38^ In terms of adverse effects, whether diuretics are an independent risk factor for CKD progression remains controversial. However, these medicines played important roles in both our models.^39–41^ Therefore, diuretics should be used with caution in patients with CKD stages 3–5. Finally, the GFR decline rate is also influenced by some immutable patient factors. The Kidney Disease Outcomes Quality Initiative guideline has provided ample evidence that African-American race (not justified in this study), male gender and older age are related to a more rapid GFR reduction.^20^ In summary, our models take advantage of the important factors involved in the progression of CKD, are consistent with current clinical practice guidelines and are highly applicable. They could be a good screening tool to determine the likelihood of initiating long-term dialysis by using the available clinical data on the patient. Several limitations need to be addressed. First, due to the patient’s lack of weight and height, the body surface area was not adjusted for eGFR. As a result, the determination of G-stage using unadjusted eGFR may be inaccurate for oversized patients. Second, using the decline in eGFR between baseline eGFR and previous eGFR may not accurately capture the progression of CKD when compared with the annual decline in eGFR during the follow-up period. Consequently, this factor did not significantly contribute to our model. Third, we only used retrospective data from three hospitals in Taipei to create our models, and it is widely recognised that racial and regional variables also influence CKD progression. Further work should involve training and validating the models through multinational and multiracial data before the clinical application is generalised. Fourth, we incorporated all important features into the prediction model, acknowledging that this approach might not be practical for clinical implementation. Nevertheless, these features underwent meticulous screening and hold varying degrees of significance in relation to CKD progression. Additionally, we assessed the model using only the top 10 important features and obtained comparable results (online supplemental tables S4 and S5, online supplemental figures S1 and S2).

## Conclusion

We have shown that using the machine learning approach can develop a highly predictive model for estimating the timing of maintenance dialysis initiation in patients with CKD stages 3–5, which provides a further step towards personalised treatment in this population.

## Data Availability

Data may be obtained from a third party and are not publicly available.
