# Supplementary material for: Personalised prediction of maintenance dialysis initiation in patients with chronic kidney disease stages 3–5: a multicentre study using the machine learning approach
Source: BMJ Health Care Inform. 2024 Apr 27;31(1):e100893. doi: 10.1136/bmjhci-2023-100893 (PMC11057266; doi:10.1136/bmjhci-2023-100893)
Supplement: Supplementary data [file bmjhci-2023-100893supp001.pdf]

Personalized Prediction of Maintenance Dialysis Initiation in Patients with Chronic Kidney Disease Stage 3-5: A Multi-Center Study using the Machine Learning Approach

Supplementary Appendix

S1. Methods 2

Table S1. Procedure-related-codes provided by Taiwan National Health Insurance 4

Table S2. Medication codes by WHO Anatomical Therapeutic Chemical......................................5

Table S3. Albuminuria Categories According to KDIGO Classification 6

Table S4. Rerun the model with including only limited number of features (top 10 important features) for 1-year model: 6

Figure S1. Rerun the model with including only limited number of features (top 10 important features) for 1-year model...7

Table S5. Rerun the model with including only limited number of features (top 10 important features) for 3-year model...7

Figure S2. Rerun the model with including only limited number of features (top 10 important features) for 3-year model...8

S1. Methods

Development of the Algorithms

ML algorithms, including logistic regression (LR), linear discriminant analysis (LDA), gradient boosting machine (GBM), LightGBM (LBGM), AdaBoost, random forest, extreme gradient boosting (XGBoost), and artificial neural network (ANN) algorithms, were used for model training and validation.

Logistic regression: Among the options in the latter category, one of the popular models in medicine is LR. An LR model calculates the class membership probability for one of two categories in a data set (Dreiseitl & Ohno-Machado, 2002). The parameters for modeling in this study were penalty = ‘l2’ and C = 0.0001.

Linear Discriminant Analysis (LDA). LDA is generally used to classify patterns between two classes; however, it can be extended to classify multiple patterns. LDA assumes that all classes are linearly separable, and according to this, multiple linear discrimination functions representing several hyperplanes in the feature space are created to distinguish the classes. If there are two classes, then LDA draws one hyperplane and projects the data onto this hyperplane to maximize the separation of the two categories (Sarraf & Pattnaik, 2020). LDA parameters were established to solve for recommended data with a large number of features using the least squares solution (lsqr), which can be combined with shrinkage or a custom covariance estimator with fixed shrinkage at 0 value.

Gradient Boosting Machine: Gradient boosting of regression trees produces competitive, highly robust, interpretable procedures for regression and classification. The ability of TreeBoost procedures to provide a quick indication of potential predictability, coupled with its extreme robustness, makes it a useful preprocessing tool that can be applied to imperfect data (Friedman, 2001). GBM was applied with default parameters and n_estimators, which is the number of boosting stages to perform at 100.

LightGBM: LGBM is a gradient-boosting framework that uses tree-based learning algorithms. It is designed to be distributed and efficient with the following advantages: faster training speed and higher efficiency, lower memory usage, better accuracy, support of parallel, distributed, and GPU learning, and capability to large-scale handling data (Microsoft, 2022). The hyper-tuning parameters of LGBM were as follows: objective = 'binary', class_weight = 'balanced', learning_rate = 0.05, reg_alpha = 0.1, reg_lambda = 0.1, subsample = 0.8, and random_state = 12.

AdaBoost: The AdaBoost algorithm is an iterative procedure that combines many weak classifiers to approximate the Bayes classifier $C*(x)$. Starting with the unweighted training sample, AdaBoost builds a classifier, such as a classification tree that produces class labels. If a training data point is misclassified, the weight of that data point is increased. A second classifier is built using the new weights, which are no longer equal. Again, misclassified training data have their weights boosted, and the procedure is repeated (Hastie et al., 2009).

Random Forest: Random Forest fits many classification trees into a dataset and then combines the predictions from all the trees. The algorithm begins with the selection of bootstrap samples from the data. Observations in the original dataset that do not occur in a bootstrap sample are

called out-of-bag observations. A classification tree fits each bootstrap sample, but at each node, only a small number of randomly selected variables are available for binary partitioning. The trees are fully grown, and each is used to predict the out-of-bag observations. The predicted observation class is calculated by the majority vote of the out-of-bag predictions for that observation, with ties split randomly (Cutler et al., 2007).

Extreme Gradient Boosting (XGBoost): XGBoost, an efficient and scalable implementation of the gradient boosting framework developed by Friedman, is an ML system for tree boosting. The scalability of XGBoost is due to several important systems and algorithmic optimizations. These innovations include a novel tree learning algorithm for handling sparse data; a theoretically justified weighted quantile sketch procedure enables handling instance weights in approximate tree learning. Parallel and distributed computing make learning faster, which enables quicker model exploration (Chen & Guestrin, 2016).

Artificial neural networks: The ANN algorithm is one of the soft computing techniques that simulates the behavior of the human brain and has become popular in its applications in data mining, fault detection, image processing, pattern recognition, weather forecasting, job scheduling, and medical diagnosis. ANNs are capable of learning through examples (train data), remembering past experiences, and performing parallel processing. Learning activities in these types of networks are possible because the neurons receive and process the information similar to the human brain (Kumar & Manjula, 2019). The model is established with the following parameters: number of hidden layers: 3, number of hidden units: 16, max. iterations: 100 with early stopping monitoring by loss function.

Table S1. Procedure-related-codes provided by Taiwan National Health Insurance

**Procedure**	**Taiwan National Health Insurance codes**
Hemodialysis (HD)	58001C, 58019C, 58020C, 58021C, 58022C, 58023C, 58024C, 58025C, and 58029C
Peritoneal dialysis (PD)	58002C, 58009A, 58009B, 58010A, 58010B, 58011A, 58011AB, 58011B, 58011C, 58012A, 58012B, 58017B, 58017C, 58026C, and 58028C
Kidney transplant	76020A, 76020B, 97416K, 97417A, and 97418B

Table S2. Medication codes by WHO Anatomical Therapeutic Chemical

ATC code	ATC name
A02A	Antiacids
A02BA	H2-receptor antagonists
A02BC	Proton pump inhibitors
A06AB	Laxatives
A10A	Insulins and analogues
A10BB	Sulfonylureas
A10BH	Dipeptidyl peptidase 4 (DPP-4) inhibitors
B03BB	Folic acid and derivatives
B01AC	Antiplatelets
C03	Diuretics
C01DA	Nitrates
C04AD	Purine derivatives
C07	Beta-blockers
C08	Calcium channel blockers
C09	Renin angiotensin blocker
C10AA	Statins
H02	Corticosteroid
J01C, J01D	Beta lactam antibiotics
M01A	Non-Steroid
M04A	Antigout preparations
R05	Cough and cold preparations
R06A	Antihistamines

Note: ATC, Anatomical Therapeutic Chemical

Table S3. Albuminuria Categories According to KDIGO Classification

	KDIGO CLASSIFICATION EQUIVALENT		
	**Normal to Mildly Increased (A1)**	**Moderately Increased (A2)**	**Severely Increased (A3)**
AER			
µg/min	<20	20–200	>200
mg/24 hours	<30	30–300	>300
ACR			
mg/g	<30	30–299	>300
mg/mmol	<3	3–30	>30
PER (mg/24 hours)	<150	150–500	>500
PCR			
mg/g	<150	150–500	>500
mg/mmol	<15	15–50	>50
Protein reagent strip	Negative to trace	Trace to +	+ or greater

Note: ACR, urinary albumin-to-creatinine ratio; AER, albumin excretion rate; KDIGO, Kidney Disease: Improving Global Outcomes; PCR, protein-to-creatinine ratio; PER, protein excretion rate.

Table S4. Rerun the model with including only limited number of features (top 10 important features) for 1-year model:

**Classifiers**	Training **AUC**	Testing **AUC**	**Accuracy**	**Sensitivity**	**Specificity**	**PPV**	**NPV**	**F1-score**
**LogisticRegression**	0.91	0.89	0.75	0.91	0.73	0.32	0.98	0.67
**LinearDiscriminantAnalysis**	0.90	0.88	0.79	0.82	0.79	0.35	0.97	0.59
**GradientBoostingClassifier**	0.96	0.88	0.80	0.84	0.79	0.36	0.97	0.59
**LGBMClassifier**	1.00	0.86	0.77	0.82	0.76	0.33	0.97	0.58
**AdaBoostClassifier**	0.95	0.83	0.73	0.83	0.72	0.29	0.97	0.54
**RandomForestClassifier**	1.00	0.87	0.83	0.78	0.83	0.40	0.96	0.59
**XGBClassifier**	1.00	0.84	0.78	0.76	0.79	0.33	0.96	0.54
**ANN**	0.98	0.96	0.88	0.87	0.77	0.34	0.98	0.49

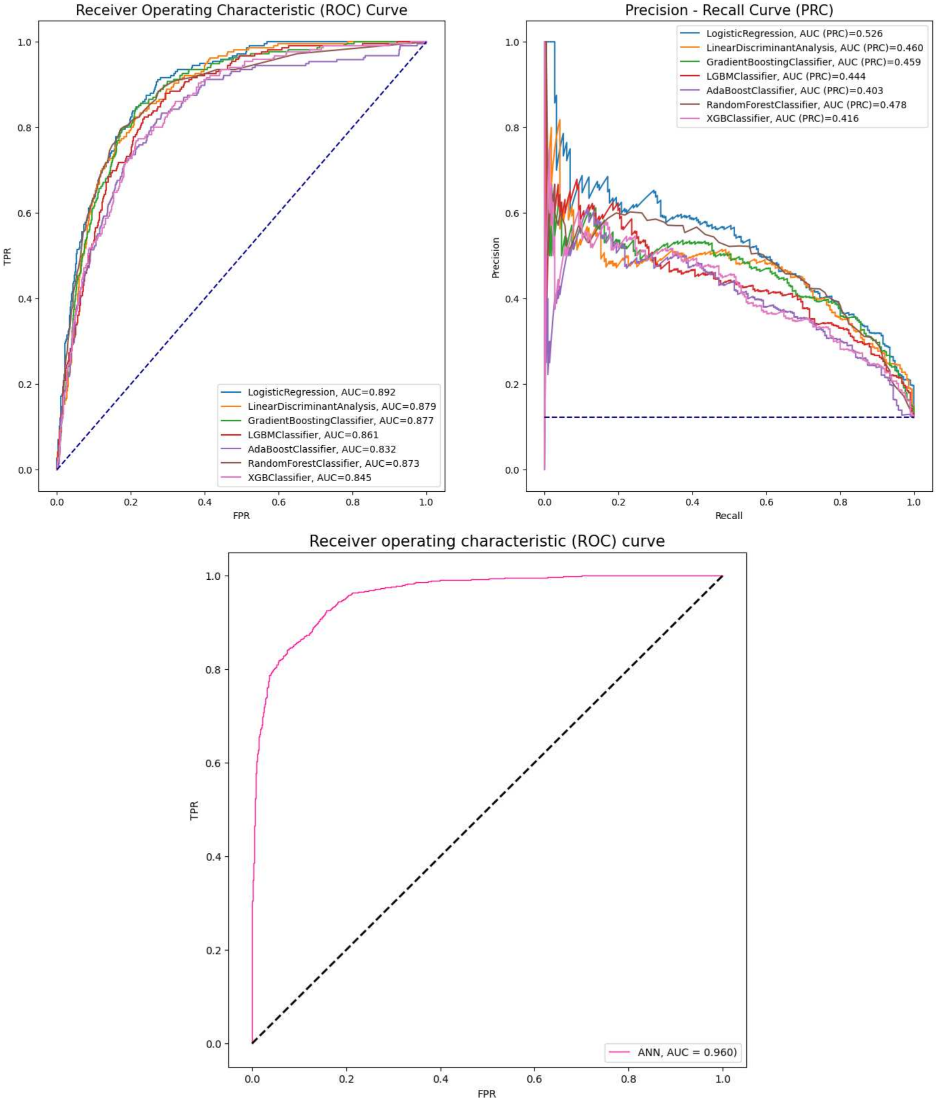

Figure S1. Rerun the model with including only limited number of features (top 10 important features) for 1-year model

Table S5. Rerun the model with including only limited number of features (top 10 important features) for 3-year model

Classifiers	Training AUC	Testing AUC	Accuracy	Sensitivity	Specificity	PPV	NPV	F1-score
**LogisticRegression**	0.89	0.88	0.79	0.85	0.78	0.53	0.94	0.74
**LinearDiscriminantAnalysis**	0.89	0.88	0.79	0.83	0.78	0.53	0.94	0.72
**GradientBoostingClassifier**	0.93	0.88	0.77	0.85	0.75	0.50	0.94	0.75

**LGBMClassifier**	1.00	0.86	0.79	0.81	0.78	0.52	0.93	0.70
**AdaBoostClassifier**	0.91	0.87	0.78	0.86	0.75	0.51	0.95	0.72
**RandomForestClassifier**	1.00	0.87	0.79	0.83	0.78	0.53	0.94	0.73
**XGBClassifier**	1.00	0.85	0.76	0.81	0.74	0.48	0.93	0.69
**ANN**	0.95	0.92	0.84	0.79	0.82	0.56	0.93	0.66

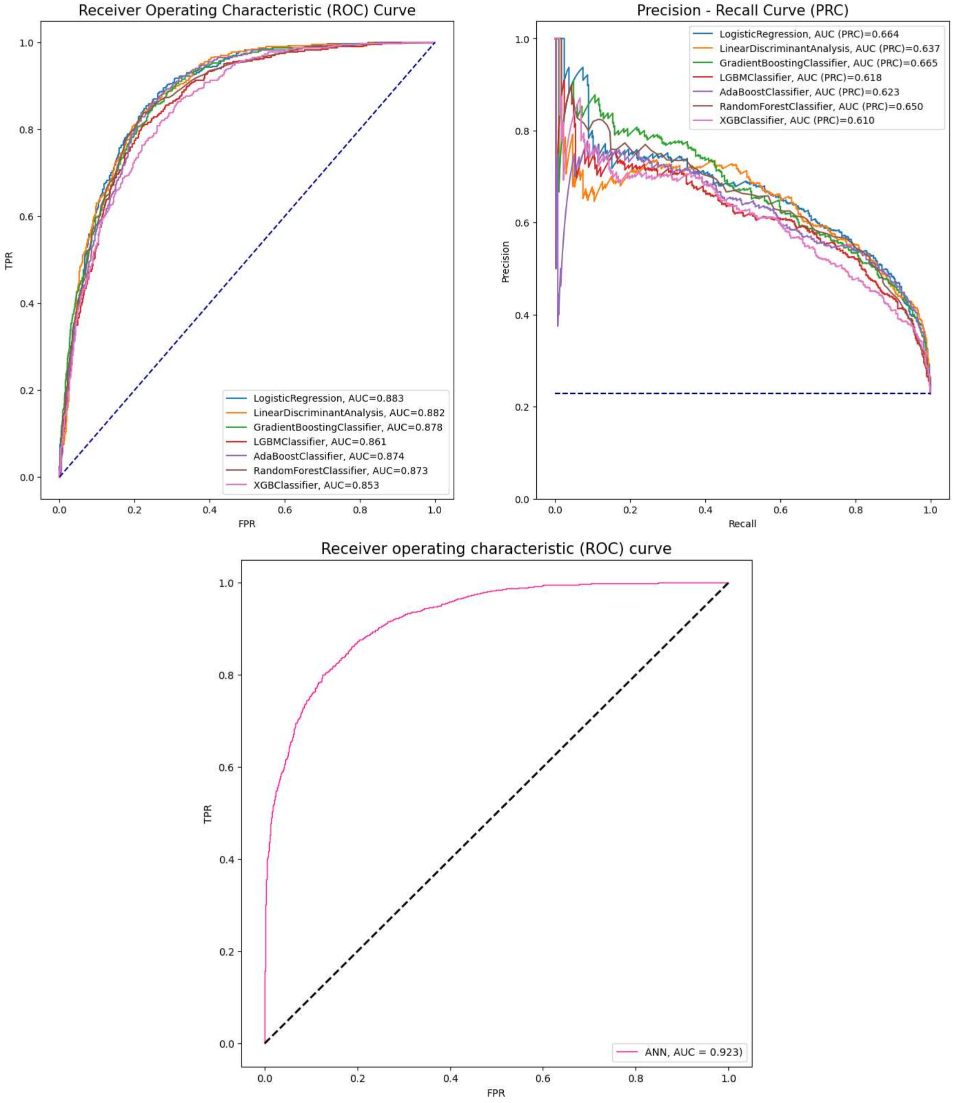

Figure S2. Rerun the model with including only limited number of features (top 10 important features) for 3-year model
